# Translation and validation of the EORTC brain cancer module (EORTC QLQ-BN20) for use in Iran

**DOI:** 10.1186/1477-7525-10-54

**Published:** 2012-05-20

**Authors:** Alireza Khoshnevisan, Mir Saeed Yekaninejad, Shahab Kamali Ardakani, Amir H Pakpour, Azam Mardani, Neil K Aaronson

**Affiliations:** 1 Department of neurosurgery, Shariati hospital, Tehran University of medical sciences, Tehran, Iran; 2 Department of Epidemiology and Biostatistics, School of Public Health, Tehran University of Medical Sciences, Tehran, Iran; 3 Qazvin Research Center for Social Determinants of Health, Qazvin University of Medical Sciences, Qazvin, Iran; 4 Department of Public Health, Qazvin University of Medical Sciences, Qazvin, Iran; 5 Brain and Spinal Injury Research Center (BASIR), Imam Khomeini Hospital Complex, Tehran University of medical sciences, Tehran, Iran; 6 Division of Psychosocial Research & Epidemiology, The Netherlands Cancer Institute, Plesmanlaan 121, 1066 CX, Amsterdam, the Netherlands

**Keywords:** Quality of life, Brain cancer, QLQ-BN20, QLQ-C30, Psychometric evaluation

## Abstract

**Background:**

The aim of this study was to translate the EORTC quality of life questionnaire for brain cancer, the QLQ-BN20, into Persian, and to evaluate its psychometric properties when used among brain cancer patients in Iran.

**Methods:**

A standard backward and forward translation procedure was used to generate the Persian language version of the QLQ-BN20. The QLQ-BN20 was administered together with the QLQ-C30 to 194 patients diagnosed with primary brain cancer. Multitrait scaling and confirmatory factor analysis (CFA) were used to evaluate the hypothesized scale structure of the questionnaire. Internal consistency reliability was estimated with Cronbach’s alpha. The ability of the QLQ-BN20 to distinguish between patient subgroups formed on the basis of performance status and cognitive status was evaluated, as was the responsiveness of the questionnaire to changes in performance status over time.

**Results:**

Multitrait scaling and CFA results confirmed the hypothesized scale structure. The measurement model was consistent across men and women. Internal consistency reliability of the multi-item scales ranged from 0.74 to 0.89. The QLQ-BN20 distinguished clearly between patients with relatively good versus poor performance and cognitive status, and changes in scores over time reflected changes observed in performance status ratings.

**Conclusions:**

These results support the validity and reliability of the QLQ-BN20 for use among Iranian patients diagnosed with primary brain cancer. Future studies should examine the psychometrics of the questionnaire when used in patients with brain metastasis.

## Background

It is estimated that 22,020 patients are diagnosed annually with brain cancer in the United States [1]. In Iran, brain cancer is the 11th most common cancer, with an annual incidence rate of approximately 550 cases for males and 800 for females [2]. Although brain cancer is relatively rare, it is a disease with serious symptoms and a poor prognosis. [1,3]. The 5-year relative survival rate is 36% [1].

There is no definite cure for patients with brain cancer. Therefore, the primary aim of treatment is to prolong the patient's life and to palliate symptoms [4]. The treatment for the most common brain tumors, gliomas, includes neurosurgery, followed by adjuvant radiotherapy, chemotherapy or combined chemoradiotherapy. All of these treatments may cause significant complications or have toxic side-effects [5,6]. Therefore, in order to select the best treatment for a specific patient it is necessary to take into consideration the stage of the disease, any risk associated with the given treatment and the patient's general health. An assessment of patient-reported outcomes, and more specifically health-related quality of life (HRQOL), is very important in this respect.

Two well-known, generic questionnaires for assessing the HRQOL of patients with cancer include The European Organization for Research and Treatment of Cancer Core Questionnaire (the EORTC QLQ-C30) and the Functional Assessment of Cancer Therapy-General (FACT-G) [7,8]. These generic questionnaires are applicable to all types of patients with cancer. Both the EORTC and the FACT measurement systems supplement their core questionnaire with condition-specific questionnaires designed to capture functional limitations and symptoms experienced by specific populations of patients. Both the EORTC and the FACT systems have developed questionnaires specific to brain cancer. The EORTC brain cancer-specific questionnaire, the QLQ-BN20, has been validated and has been translated into a number of primarily European languages [9,10]. The aim of the present study was to translate the QLQ-BN20 into the dominant language in Iran, Persian (spoken by approximately two-thirds of the population), and to generate psychometric data regarding the questionnaires reliability and validity when used among Iranian brain cancer patients.

## Methods

### Translation of the QLQ-BN20

The QLQ-BN20 questionnaire contains 20 items organized into four scales; future uncertainty (3 items), visual disorder (3 items); motor dysfunction (3 items); and communication deficit (3 items), and seven single items (headaches, seizures, drowsiness, hair loss, itchy skin, weakness of legs, and bladder control) [9,10]. All items are rated on a four-point Likert-type scale (‘not at all’, ‘a little’, ‘quite a bit’ and ‘very much’), and are linearly transformed to a 0-100 scale, with higher scores indicating more severe symptoms [11].

We obtained permission from the EORTC Quality of Life Department to generate the Iranian version of the QLQ-BN20. The aim of the translation procedure was to provide an Iranian version of the questionnaire that was conceptually equivalent to the original English language version.

We used the standard EORTC translation procedures [12]. Briefly, two trained bilingual Iranians independently translated the QLQ-BN20 into Persian. A specialized translator (project manager; AHP) compared these two versions, evaluated the level of difficulty in terms of translation as well as the equivalence of each item and response scale. The translators compared their translations, reconciled their discrepancies and arrived at a unified Persian version of the questionnaire. Subsequently, the provisional Persian version was independently translated back into U.S. English by two additional translators who were native English speakers. The Project Manager compared the English translations with the original questionnaire, and any discrepancies between the back-translations were resolved with the translators. This process went through two iterations, and the documentation was reviewed by the EORTC Quality of Life Group. The resulting Persian version of the QLQ-BN20 was administered to 10 brain tumor patients (6 women and 4 men; age range = 28–69 years). All patients were able to complete the questionnaire without assistance, and reported that the questions were clear and easy to complete.

### Validation of the Persian version of the QLQ-BN20

Study participants were patients with primary brain cancer who had been referred to Shariati Hospital and the Cancer Institute of Iran in Tehran. These centers are national referral centers for cancer treatment in Iran. Inclusion criteria were; a histologically verified diagnosis of brain cancer, older than 18 years, fluency in Persian, and a life expectancy of at least 4 weeks. All patients provided written informed consent. Patients with a previous cancer diagnosis, those who were too frail and those who were unable to understand the questionnaire or unwilling to provide written informed consent, were excluded. The study protocol was approved by the Ethical Committee of Tehran University of Medical Sciences. All participants gave their written informed consent.

#### Data collection

Patients were recruited into the study after surgery and before chemotherapy (CT), radiotherapy (RT) or chemoradiotherapy (CRT). Patients completed the validated Persian version of the QLQ-C30 [13] and the Persian version of the QLQ-BN20 before the start of the CT, RT or CRT, and again approximately four weeks later. We obtained sociodemographic and clinical information via questionnaire and medical record review. This included age, sex, education, tumor type, and surgery. Additionally, the Mini-Mental State Examination (MMSE) and The Karnofsky Performance Status Scale (KPS) were administered to all patients.

The questionnaire was completed by interview if the patients were illiterate. The interviewer read the questions to the patients without embellishment or explanation, and presented the possible responses and elicited patients’ choices without coaching.

#### Statistical analysis

Ceiling and floor effects were calculated for all QLQ- BN20 scales. A ceiling or floor effect lower than 15% indicates acceptable measurement standards [14].

We evaluated the scale structure of the QLQ-BN20 in several ways. First, we used multitrait scaling to assess item convergent and discriminant validity. We calculated Spearman correlation coefficients between each item and its own scale, corrected for overlap. Item-scale correlations of 0.40 or greater were taken as evidence of item convergent validity [15]. We also calculated the correlations between each item and the other scales. We expected that items would correlate more highly (two times the standard error, 1/√N) with their own scale than with other scales.

Second, we used confirmatory factor analysis (CFA) to test the hypothesized model fit. We employed three categories of indices: absolute fit indices, incremental fit indices and parsimony fit indices. An absolute fit index assesses how well an *a priori* model reproduces the sample data. We used chi-square, root mean square error of approximation (RMSEA) and standardized root mean square residual (SRMR) as absolute fit indices [16,17]. Chi-square assesses the magnitude of discrepancy between the sample and fitted covariance matrices [18]. However, the chi-square statistic is sensitive to sample size and therefore with large samples, is not a practical test of model fit. RMSEA incorporates a penalty function for poor model parsimony [16]. RMSEA, values in the range of 0.05–0.08 were taken to indicate acceptable fit, values in the range of 0.08–0.10 to indicate marginal fit, and values larger than 0.10 to indicate poor fit [16]. SRMR is the square root of the difference between the residuals of the sample covariance matrix and the hypothesized covariance model. Values of the SRMR less than 0.10 are generally considered favorable.

We used the non-normed fit index (NNFI, also known as the Tucker-Lewis index) and the comparative fit index (CFI) to estimate incremental fit. The suggested cut off for NNFI and CFI is 0.90 or greater.

We computed the parsimonious normed fit index (PNFI) to assess the parsimony the model [17,19]. For PNFI we did not employ any absolute standard of model fit, but rather simply noted that higher PNFI values reflect more parsimonious fit [19]. We used weighted least squares (WLS) as the method of estimation.

In addition, we examined the model invariance across gender. To compare the factor loadings across gender, we applied multi-group measurement invariance analysis. There are different terminologies in the literature for tests of invariance. We used two terms of factorial invariance (i.e., configural invariance and metric invariance). In configural invariance, the pattern of fixed- and free-factor loadings is constant across groups, while the magnitudes of these loadings are not constrained to be equal. For metric invariance, the magnitudes of factor loadings for particular items are invariant across groups [20]. As suggested by Cheung, differences in practical fit indices such as CFI and NNFI not larger than 0.01 between NNFI or CFI values were considered as evidence of model invariance [20]. We hypothesized that the Persian version of QLQ-BN20 would perform similarly across gender.

We estimated internal consistency reliability of the QLQ-BN20 scales with Cronbach's coefficient alpha. An alpha coefficient of 0.70 or higher was considered acceptable for purposes of group comparisons [21].

“Known groups” or clinical validity was evaluated by comparing the QLQ-BN20 scores between patients grouped according to KPS and MMSE scores. We hypothesized that patients with higher KPS (>80) would score better on the QLQ-BN20 than those with lower KSP (≤80) [9,10]. We also anticipated that patients with higher MMSE (≥27) would score better on the QLQ-BN20 than those with lower MMSE (<27) [10]. Analysis of covariance (ANCOVA) was used to test for group differences. To control for probability of type I errors due to multiple comparisons, we used an adjustment procedure developed by Benjamini and Hochberg. This procedure controls the false discovery rate. The false discovery rate level was set at 5% [22,23]. Effect sizes (Cohen’s d statistic) were calculated to estimate the magnitude of observed, statistically significant group differences [24].

Finally, to evaluate the responsiveness of the QLQ-BN20 to change in health status over time, we classified patients as having worse, stable or improved KPS scores from baseline to follow-up. We evaluated changes in QLQ-BN20 scores as a function of change in KPS with analysis of covariance (ANCOVA), adjusting for baseline values.

We used SPSS 16.0 for Windows and LISREL 8.8 for data analyses. A p value <0.05 was considered as statistically significant.

## Results

In total, 194 patients were recruited into the study. The patients' baseline characteristics are provided in Table 1. The mean age of the patients was 42 ± 5 years, with a range between 18 and 80 years. Forty-seven percent of the patients was female. The majority of patients (66%) was married, and 46% had completed secondary school or college.

**Table 1:** Sociodemographic and clinical characteristics of the study sample (n = 194)*

	**Total**
**Age (years) >Mean (SD)**	42.5 (16.1)
**Sex**	
Male	103 (53.0%)
Female	91 (47.0%)
**Educational status**	
Illiterate	35 (18.0%)
Primary school	33 (17.0%)
Middle school	36 (18.6%)
Secondary school	48 (24.8%)
College	42 (21.6%)
**Marital status**	
Single	43 (22.2%)
Married	128 (65.9%)
Widowed/divorced	23 (11.9%)
**KPS**	
≤80	102 (52.5%)
>80	92 (47.5%)
**MMSE**	
Mean (SD)	23.89(5.3)
Median	26.00
Range	8-30
**Type of surgery**	
Biopsy only	40 (20.6%)
Partial resection	96 (49.5%)
Total resection	58 (29.9%)
**Type of adjuvant therapy**	
CT	75(38.7%)
RT	62 (32%)
CT + RT	57 (29.3%)
**Tumor type**	
Astrocytoma	72(37.1%)
Atypical Meningioma*	46(23.7%)
Oligodendroglioma	53(27.3%)
Others	23(11.9%)

***KPS*** **= Karnofsky Performance Status;*MMSE*** **= Mini-Mental State Examination**.

*Patients with grade 2 and 3 tumors including, chordoid, atypical, clear cell, rhabdoid, anaplastic (malignant) and papillary subtypes. Meningeal sarcomas (angioblastic meningiomas) and hemangiopericytomas were also included in the Atypical Meningioma.

Astrocytomas (37.1%) constituted the most frequent tumor type. Slightly more than one-third of patients were receiving CT, one-third RT, and slightly less than one-third CT + RT. Only seven patients (3.65%) were lost to follow-up assessment due to death.

As shown in Table 2, all subscales of the QLQ-BN20 were found to be normally distributed (Kolmogorov-Smirnov, p >0.05). There was no ceiling effect for any of the BN20 subscales. However, some floor effects were observed for seizures, headaches, itchy skin and bladder control.

**Table 2:** Descriptive statistics for the QLQ-BN20

		**Number of forms**	**Mean (SD)**	**Floor N (%)**	**Ceiling (%)**	**Cronbach’s alpha**	**Normality (Kolmogoroff-Smirnoff-Test)**
BFU (future uncertainty)	Baseline	194	39.9 (24.9)	8 (4.1%)	3 (1.5%)	0.80	0.14
	Follow-up	187	36.8 (24.5)	10 (5.3%)	3 (1.6%)	0.74	0.24
BVD (visual disorder)	Baseline	194	27.9 (26.9)	17 (8.7%)	5 (2.6%)	0.74	0.16
	Follow-up	187	25.8 (25.9)	14 (7.4%)	4 (2.1%)	0.80	0.15
BMD (motor dysfunction)	Baseline	194	29.0 (28.9)	28 (14.4%)	6 (3.1%)	0.80	0.46
	Follow-up	186	28.6 (29.6)	23 (12.3%)	3 (1.6%)	0.82	0.18
BCD (communication deficit)	Baseline	194	23.7 (27.2)	18 (9.2%)	3 (1.5%)	0.89	0.21
BHA (headaches)	Follow-up	187	20.9 (25.0)	14 (7.5%)	1 (0.5%)	0.89	0.22
	Baseline	192	46.9 (33.9)	25 (13.0%)	24 (12.5%)	-	0.19
	Follow-up	187	43.6 (35.1)	21 (11.2%)	18 (9.6%)	-	0.15
BSE (seizures)	Baseline	194	10.0 (21.6)	99 (51.5%)	7 (3.6%)	-	0.13
	Follow-up	186	7.7 (20.0)	95 (51.0%)	1 (0.5%)	-	0.11
BDR (drowsiness)	Baseline	192	32.2 (31.7)	28 (14.6%)	11 (5.7%)	-	0.6
	Follow-up	185	31.2 (32.0)	19 (10.3%)	8 (4.3%)	-	0.5
BHL (hair loss)	Baseline	194	23.8 (32.5)	41 (21.1%)	9 (4.6%)	-	0.28
	Follow-up	187	25.9 (44.4)	38 (20.3%)	10 (5.3%)	-	0.06
BIS (itchy skin)	Baseline	193	14.0 (25.8)	54 (28.0%)	5 (2.6%)	-	0.20
	Follow-up	186	17.8 (27.5)	46 (24.7%)	6 (3.2%)	-	0.32
BWL (weakness of legs)	Baseline	190	28.4 (33.9)	23 (12.1%)	14 (7.4%)	-	0.36
BBC (bladder control)	Follow-up	187	28.3 (34.1)	21 (11.2%)	12 (6.4%)	-	0.29
	Baseline	194	15.1 (25.8)	67 (34.5%)	4 (2.0%)	-	0.20
	Follow-up	186	15.2 (30.3)	58 (31.2%)	6 (3.2%)	-	0.30

Results of the multitrait scaling analysis are presented in Table 3. All items correlated 0.60 or higher with their own scale, corrected for overlap. There were no scaling errors, with all items correlating two standard errors or higher with their own scales than with other scales.

**Table 3:** Results of the multitrait scaling analysis*

	**BFU**	**BVD**	**BMD**	**BCD**
**BFU (future uncertainty)**				
uncertain about the future	**0.75**	0.15	0.04	0.19
setbacks in condition	**0.62**	0.06	0.10	0.28
disruption of family life	**0.78**	0.23	0.10	0.17
future worsen	**0.67**	0.21	0.18	0.04
**BVD (visual disorder)**				
double vision	0.14	**0.67**	0.11	0.18
vision blurred	0.08	**0.86**	0.02	0.07
difficulty reading	0.16	**0.83**	0.10	0.05
**BMD (motor dysfunction)**				
weakness on one side of body	0.10	0.25	**0.79**	0.15
trouble with coordination	0.01	0.12	**0.80**	0.19
feel unsteady on your feet	0.18	0.12	**0.86**	0.18
**BCD (communication deficit)**				
Trouble finding the right words to express yourself	0.14	0.21	0.11	**0.88**
Difficulty speaking	0.13	0.13	0.12	**0.88**
Trouble communicating thoughts	0.09	0.23	0.13	**0.87**

* Correlations are based on analysis of the baseline data.

**Figure 1:**
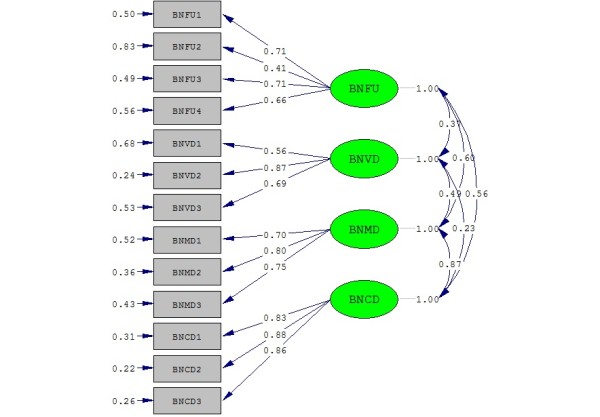
Four-factor structure of the QLQ-BN20.

Results of the CFA analysis indicated that all of the goodness of fit indices supported the four-factor model for the QLQ-BN20: *χ*2 = 92.51, degree of freedom = 59, p <0.001, CFI = 1.0, RMSEA = 0.068, SRMR = 0.066, NNFI = 1.0, and PNFI = 0.73. Moreover, all factor loadings were significant, ranging from 0.24 to 0.89 (Figure 1). Additional analyses were based on a four-factor model that constrained the factor loadings to be equal across gender groups. The measurement model revealed an acceptable model fit for the QLQ-BN20: *χ*2 = 190.5, degree of freedom = 133, p <0.001, CFI = 0.923, SRMR = 0.089, NNFI = 0.911, PNFI = 0.704, RMSEA = 0.080. Moreover, metric invariance showed good fit indices: *χ*2 = 205.15, degree of freedom = 133, p <0.001, CFI = 0.927, SRMR = 0.091, NNFI = 0.915, PNFI = 0.753, RMSEA = 0.079. These results support the stability of the hypothesized factor structure across sexes.

The internal consistency reliability of all scales of the QLQ-BN20 was acceptable for group comparisons (Table 2). Cronbach's alpha coefficient for all scales was above the 0.70 threshold. Communication deficit had the highest internal consistency (0.89 at both baseline and follow-up) while the lowest was for future uncertainty (0.80, 0.74).

Results from the known group comparisons between subgroups of patients formed on the basis of performance status and MMSE are summarized in Table 4. As hypothesized, patients with better KPS and MMSE scores had significantly better scores on all of the QLQ-BN20 multi-item scales than patients with lower KPS and MMSE scores. Effect sizes (Cohen’s d statistic) ranged from 0.18 to 1.56 for the KPS, and from 0.20 to 0.65 for the MMSE.

**Table 4:** Known groups validity testing: Comparison of QLQ-BN20 Scores as a function of KPS and MMSE

		**KPS**		**Effect size**	MMSE**		**Effect size**
		**>80**	**≤80**		**<27**	**≥27**	
					**N = 91**	**N = 103**	
	**Baseline**	**N = 53,**	**N = 141,**				
	**Follow-up**	**N = 41**	**N = 153**				
BFU (future uncertainty)	Baseline*‡	42.7 (22.2)	33.5 (24.7)	0.38	33.9 (24.4)	27.2 (23.4)	0.28
	Follow-up *	37.0 (15.1)	25.6 (24.5)	0.50	-	-	-
BVD (visual disorder)	Baseline *‡	38.3 (30.1)	27.0 (27.3)	0.40	28.1 (29.4)	21.8 (22.8)	0.24
	Follow-up *	33.3 (27.8)	23.7 (26.1)	0.36	-	-	
BMD (motor dysfunction)	Baseline*‡	68.2 (31.8)	24.6 (26.3)	1.56	33.7 (30.2)	16.5 (22.5)	0.65
	Follow-up *	63.9 (29.1)	22.4 (27.0)	1.5	-	-	
BCD (communication deficit)	Baseline *‡	44.9 (31.9)	20.3 (26.5)	0.88	26.1 (28.3)	14.1 (21.8)	0.48
	Follow-up*	45.0 (33.4)	17.0 (22.6)	1.11	-	-	
BHA (headaches)	Baseline *‡	50.0 (36.0)	44.3 (32.2)	0.17	47.8 (28.6)	34.8 (34.4)	0.41
	Follow-up *	63.3 (36.7)	42.5 (35.1)	0.59	-	-	
BSE (seizures)	Baseline *‡	15.1 (22.9)	11.0 (23.3)	0.18	14.3 (25.5)	5.7 (15.6)	0.41
	Follow-up *	14.8 (24.2)	8.5 (21.2)	0.29	-	-	
BDR (drowsiness)	Baseline *‡	57.6 (30.1)	28.3 (31.2)	0.95	34.7 (33.3)	22.3 (25.0)	0.42
	Follow-up *	66.7 (27.2)	26.6 (30.5)	1.34	-	-	
BHL (hair loss)	Baseline*‡	33.3 (35.1)	22.1 (32.2)	0.34	23.4 (32.8)	16.8 (33.5)	0.20
	Follow-up*	63.3 (36.7)	42.5 (35.1)	0.59	-	-	
BIS (itchy skin)	Baseline*‡	33.3 (35.1)	13.2 (24.7)	0.72	19.2 (30.4)	9.2 (17.6)	0.41
	Follow-up*	40.0 (37.8)	16.5 (26.5)	0.80	-	-	
BBC (bladder control)	Baseline*‡	45.4 (37.3)	12.8 (24.2)	1.15	19.0 (27.3)	9.2 (23.4)	0.39
	Follow-up*	50.0 (42.3)	12.9 (28.2)	0.97	-	-	

* Statistically significant for KPS.

‡ Statistically significant for MMSE.

** MMSE was only available at baseline.

Finally, changes in QLQ-BN20 scores over time as a function of changes in KPS scores (worse, stable or improved performance status) are reported in Table 5. The results supported the ability of all of the QLQ-BN20 scales to detect such shifts in KPS, with the exception of seizures, drowsiness, visual disorder and hair loss which did not vary as a function of change in KPS.

**Table 5:** Changes of QLQ-BN20 over time by performance status

	**KSP**			
	**Worsening**	**Stable**	**Improved**	**P value (ANCOVA)**
	**N = 59**	**N = 49**	**N = 79**	
	**Mean (SD)**	**Mean (SD)**	**Mean (SD)**	
Headaches ^*^	12.4 (29.2)	1.2 (35.4)	-10.0 (32.6)	0.04
Motor dysfunction^*^	6.1 (18.8)	0.1 (18.2)	-4.3 (15.4)	0.01
Drowsiness	5.5 (23.2)	2.4 (27.3)	1.7 (24.3)	0.38
Weakness of legs^*†^	15.2 (28.4)	-1.3 (20.2)	-12.1 (19.3)	<0.01
Visual disorder	5.4 (16.1)	2.7 (25.4)	1.6 (22.3)	0.64
Bladder control^*†^	9.6 (18.8)	-0.7 (28.9)	-6.6 (26.3)	0.01
Seizures	5.3 (19.8)	3.2 (21.8)	2.7 (17.6)	0.72
Hair loss	12.4 (29.2)	-4.5 (50.4)	-15.2 (45.4)	0.08
Itchy skin^*^	6.5 (20.8)	0.4 (26.4)	-3.8 (24.8)	0.02

* P < 0.05 Improved Vs. Worsening Group.

† P < 0.05 Stable Vs. Worsening Group.

## Discussion

In this paper we have reported on the results of the translation and validation of the Persian version of the QLQ-BN20 for use with primary brain cancer patients in Iran. This included examination of the hypothesized scale structure of the questionnaire, internal consistency reliability estimation, known groups validity testing, and evaluation of the responsiveness of the QLQ-BN20 to changes over time in health status.

Overall, the results lend strong support to the psychometric robustness of the questionnaire. The large majority of patients completed all items and there were very minor missing items. Ceiling effects were absent while some floor effects were observed for items assessing seizures, hair loss, itchy skin and bladder control. A possible reason for this latter finding could be that most of the patients were newly-diagnosed and had only recently initiated adjuvant treatment. With longer follow-up, more variance in scores for these symptoms could be expected. Our results are in accordance with previous studies [9,10].

The results of both the mulitrait scaling and CFA provided strong support for the hypothesized scale structure of the QLQ-BN20. To our knowledge, ours is the first study to employ CFA in the evaluation of the QLQ-BN20 measurement model. Some studies have reported that females with brain tumors tend to report worse QOL than their male counterparts [25-27]. It is important to determine if these gender differences also affect the scale structure of the questionnaire. [28]. Toward this end, we assessed the factorial invariance of the QLQ-BN20. The results of the CFA demonstrated that the hypothesized four-factor model fit the data of both the male and female samples well, supporting invariance. To our knowledge, ours is the first study to assess factor structure of the QLQ-BN20 for patients with primary brain tumours using confirmatory factor analysis We would recommend that this be undertaken in other cultural and language settings as well.

The internal consistency reliability of the QLQ-BN20 scales exceeded the recommended 0.70 level, with most coefficients in the 0.80 to 0.90 range. These reliability estimates are even higher than those reported in previous studies with English-speaking and multi-national samples [9,10].

The QLQ-BN20 was able to distinguish clearly between subgroups of patients formed on the basis of their performance status and cognitive status, and was responsive to change over time in performance status. Again, these results are in line with those of previous studies [9,10].

Our study had several limitations that should be noted. First, we recruited a convenience sample of patients. Nevertheless, we believe that the sample sufficiently represents the population of interest. Second, although the overall sample size was sufficient for the analyses that we conducted, there were insufficient numbers available to carry out subgroup analyses of more homogenous groups of brain cancer patients. This is something that could be done in future studies. Finally, the study was of relatively short duration, and thus we had a relatively short period evaluate the responsiveness of the QLQ-BN20 over time. Use of the questionnaire in large, prospective studies should provide the opportunity to examine the responsiveness of the questionnaire over long periods of time.

In conclusion, the results of our study support the feasibility, measurement model, reliability and validity the QLQ-BN20 in assessing the HRQOL of Iranian patients with primary brain cancer. The availability of this questionnaire in Persian will facilitate the assessment of the health-related quality of life of brain cancer patients in Iran, particularly in the context of clinical research, but also potentially in clinical practice setting. Future studies are needed to examine the psychometric properties of the Iranian version of the QLQ-BN20 among patients with brain metastases.

## Competing interests

The authors declare that they have no competing interests.

## Authors' contributions

AKh and AHP were responsible for designing the study, analyzing and interpreting the data, and drafting the manuscript. ShKA and AM were responsible for data collection. NKA interpreted the data, and revised the manuscript. The analyses were performed by MY. All authors have read and approved the final manuscript.
